# Cavin1 Deficiency Causes Disorder of Hepatic Glycogen Metabolism and Neonatal Death by Impacting Fenestrations in Liver Sinusoidal Endothelial Cells

**DOI:** 10.1002/advs.202000963

**Published:** 2020-08-21

**Authors:** Zhuang Wei, Jigang Lei, Feng Shen, Yuxiang Dai, Yan Sun, Yilian Liu, Yan Dai, Zhijie Jian, Shilong Wang, Zhengjun Chen, Kan Liao, Shangyu Hong

**Affiliations:** ^1^ State Key Laboratory of Genetic Engineering and School of Life Sciences Human Phenome Institute Fudan University Shanghai 200433 China; ^2^ Key Laboratory of Systems Biology Innovation Center for Cell Signaling Network CAS Center for Excellence in Molecular Cell Science Institute of Biochemistry and Cell Biology Shanghai Institutes for Biological Sciences CAS 320 Yueyang Road Shanghai 200031 China; ^3^ The Department of Biology Tongji University Shanghai 200092 China; ^4^ Department of Hepatobiliary Surgery Dongfeng Hospital Hubei University of Medicine Shiyan Hubei 442001 China; ^5^ Department of Cardiology Zhongshan Hospital Fudan University Shanghai Institute of Cardiovascular Disease Shanghai 200031 P. R. China; ^6^ Masonic Medical Research Institute 2150 Bleecker St Utica NY 13501 USA; ^7^ State Key Laboratory of Cell Biology Key Laboratory of Systems Biology Innovation Center for Cell Signaling Network CAS Center for Excellence in Molecular Cell Science Institute of Biochemistry and Cell Biology Shanghai Institutes for Biological Sciences CAS 320 Yueyang Road Shanghai 200031 China; ^8^ Department of Radiology the First Affiliated Hospital of Xi'an Jiaotong University Xi'an 710049 China

**Keywords:** Cavin1, congenital generalized lipodystrophy type 4, fenestration, glycogen metabolism, liver sinusoidal endothelial cells

## Abstract

It has been reported that Cavin1 deficiency causes lipodystrophy in both humans and mice by affecting lipid metabolism. The ablation of Cavin1 in rodents also causes a significant deviation from Mendelian ratio at weaning in a background‐dependent manner, suggesting the presence of undiscovered functions of Cavin1. In the current study, the results show that Cavin1 deficiency causes neonatal death in C57BL/6J mice by dampening the storage and mobilization of glycogen in the liver, which leads to lethal neonatal hypoglycemia. Further investigation by electron microscopy reveals that Cavin1 deficiency impairs the fenestration in liver sinusoidal endothelial cells (LSECs) and impacts the permeability of endothelial barrier in the liver. Mechanistically, Cavin1 deficiency inhibits the RhoA‐Rho‐associated protein kinase 2‐LIM domain kinase‐Cofilin signaling pathway and suppresses the dynamics of the cytoskeleton, and eventually causes the reduction of fenestrae in LSECs. In addition, the defect of fenestration in LSECs caused by Cavin1 deficiency can be rescued by treatment with the F‐actin depolymerization reagent latrunculin A. In summary, the current study reveals a novel function of Cavin1 on fenestrae formation in LSECs and liver glycogen metabolism, which provide an explanation for the neonatal death of Cavin1 null mice and a potential mechanism for metabolic disorders in patients with Cavin1 mutation.

## Graphical abstract

Cavin1 deficiency causes severe hypoglycemia, dampens the storage and mobilization of glycogen in mouse liver, and results in neonatal death in C57BL/6J mice. Mechanistically, by inhibiting the dynamics of actin cytoskeleton, Cavin1 deficiency reduces the fenestrae in liver sinusoidal endothelial cells, which impairs bilateral flow between liver glycogen and blood glucose.

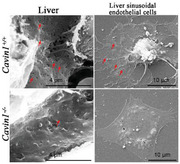

Congenital generalized lipodystrophy type 4 (CGL4) is a rare disease caused by mutations in the gene *CAVIN1*, also known as polymerase I and transcript release factor, with systemic loss of body fat and predisposition for diabetes mellitus, hypertriglyceridemia, and hepatic steatosis.^[^ ^1^, ^2^ ^]^

Cavin1 is the first found member of Cavins family, a family of proteins play important roles in the formation and maintenance of caveolae.^[^ ^3^ ^]^ The other three members in Cavins family: Cavin2 (serum‐deprivation response protein, SDPR), Cavin3 (SDR related gene product that binds to c‐kinase), and Cavin4 (muscle‐restricted coiled‐coil protein, MURC) all share homology with Caivn1 and regulate the dynamics of Caveolae.^[^ ^4^, ^5^ ^]^ Cavin1 and Caveolin1 are two important component proteins for caveolae. Lacking either one ablates caveolae completely.^[^ ^6^ ^]^ The defective caveolae will lead to diverse diseases including cancer, lipodystrophy, cardiomyopathy, and muscular dystrophies.^[^ ^6^ ^]^ Most knowledge about the functions of caveolae comes from the studies of caveolins and cavins, especially the studies of caveolin or cavin deficient mice.^[^ ^4^, ^6^, ^7^ ^]^ Most phenotypes of Caveolin1 or Cavin1 deficient mice are centered on metabolism, such as reduced adipose tissue, hyperlipidemia, glucose intolerance, hyperinsulinemia, and less physical activity.^[^ ^4^, ^6^, ^7^ ^]^

Cavin1 deficient mice show similar phenotypes as humans with severe lipodystrophy and metabolic disorders.^[^ ^8^ ^]^ Thus, Cavin1‐deficient mice are a viable representative model for studying CGL4 disease. Recent studies reported a significant deviation from Mendelian inheritance for *Cavin1* knockout pups in certain genetic background at weaning,^[^ ^9^, ^10^, ^11^ ^]^ but the cause of this deviation remains unclear. Studying the underlying mechanisms for this phenomenon may reveal novel functions of Cavin1 and provide better care for CGL4 patients.

The survival rate of the *Cavin1* knockout pups at weaning (3 weeks of age) is much lower than expected and varies from 5.7% to 13% in previous reports.^[^ ^9^, ^10^, ^11^ ^]^ A possible explanation for the variation is the mixed genomic background of the mice used in different laboratories. In order to study the cause of *Cavin1* knockout pup mortality in a pure genetic background, we backcrossed the *Cavin1* ^+/−^ mice with C57BL/6J for 20 generations. Then, we crossbred the backcrossed *Cavin1* ^+/−^ heterozygous parents to produce *Cavin1* ^−/−^ homozygous progenies. Among 72 offspring (from 15 litters) genotyped at weaning, only one was identified as *Cavin1* ^−/−^ (**Figure** **1**A and Table S1, Supporting Information). To ascertain the cause of missing adult *Cavin1* ^−/−^ mice, we analyzed 107 (from 12 litters) newborn pups from heterozygous parents. The ratio of *Cavin1* ^+/+^/*Cavin1* ^+/−^/*Cavin1* ^−/−^ at birth followed the Mendelian inheritance (Table S2, Supporting Information), suggesting that the death of *Cavin1* ^−/−^ mice happened after birth rather than at the embryonic stage. The survival survey concluded that most of the death occurred at around 10 h after birth (Figure 1B). We also observed that all the dying *Cavin1* ^−/−^ mice exhibited cyanosis (Figure 1C,D). Cyanosis is a blue color throughout the skin and mucous membranes due to reduced hemoglobin in the blood, which could be caused by multiple conditions including heart disease, respiration failure, and extreme hypoglycemia.^[^ ^12^ ^]^ Generally, cyanosis and death caused by respiration failure would occur within 2 h after birth,^[^ ^13^ ^]^ but this timeline did not match our observations where *Cavin1* ^−/−^ mice survived ≈10 h after birth (Figure 1B). Moreover, the morphology of lung and heart in *Cavin1* ^−/−^ mice was normal (Figure S1A,B, Supporting Information). These results suggested that it might not be the respiration failure or heart diseases, but the disorder of glucose homeostasis caused the neonatal death of *Cavin1* ^−/−^ mice. To test this hypothesis, we measured the blood glucose level at 9 h after birth in the pups that survived and found that the blood glucose levels in the *Cavin1* ^−/−^ pups were extremely low, while the glucose levels in other littermates remained normal (Figure 1E). To determine whether the lethal hypoglycemia in *Cavin1* ^−/−^ pups was prenatal or postnatal, we measured fetal blood glucose level at 24 h before birth. Our data showed that the *Cavin1* ^+/+^ and *Cavin1* ^−/−^ fetus had a similar level of blood glucose before birth (Figure 1F). In contrast, in the same cohort, the blood glucose level in *Cavin1* ^−/−^ pups was significantly lower than their wild‐type littermates at 9 h after birth (Figure 1F). These results indicated hypoglycemia in *Cavin1* ^−/−^ pups occurred at the postnatal stage. To test if this severe hypoglycemia led to neonatal death, we performed a rescue experiment. We found that two intraperitoneal injections of glucose at 1 and 5 h after birth rescued *Cavin1* ^−/−^ neonates (Figure 1G,H). Without any further intervention, these glucose‐treated mice survived the weaning stage like their wild‐type littermates except a leaner body (Figure 1H,I). In summary, our data demonstrated that *Cavin1* ^−/−^ mice backcrossed to the C57BL/6J background suffered severe hypoglycemia leading to neonatal death.

**Figure 1:**
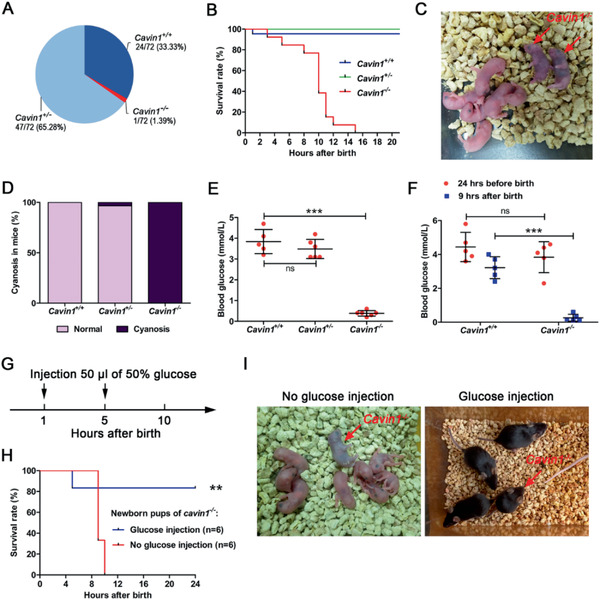
The Cavin1 deficient mice in C57BL/6J background suffered severe hypoglycemia and died at neonatal stage. A) The survival rate of newborn pups from *Cavin1* ^+/−^ parents at weaning (3 weeks). B) The survival rate of newborn pups within 21 h. *Cavin1* ^+/+^, *n* = 22; *Cavin1* ^+/−^, *n* = 34; and *Cavin1* ^−/−^, *n* = 13. C) Cyanosis of Cavin1 deficient pups at 10 h after birth. Red arrows showed Cavin1 null pups. D) The percentage of pups with cyanosis at 10 h after birth. E) Blood glucose level in *Cavin1* ^+/+^, *Cavin1* ^+/−^, and *Cavin1* ^−/−^ pups at 9 h after birth. *** *p* < 0.001; ns, not significant; *n* = 5–6 pups each group. F) Blood glucose level in *Cavin1* ^+/+^ and *Cavin1* ^−/−^ fetus at 24 h before birth and pups at 9 h after birth. *** *p *< 0.001; ns, not significant; *n* = 5 fetus or pups each group. G) Schematic diagram of rescue of newborn pups with two glucose injections at 1 and 5 h after birth. H) The survival rate of newborn *Cavin1* ^−/−^ pups intraperitoneal injected with 50 µL of 50% d‐glucose solution in phosphate buffered saline (PBS) or an equal volume of PBS. ** *p *< 0.01; *n* = 6 pups each group. I) The rescue results of newborn pups with or without glucose injection after birth. Left: Red arrow pointed the only *Cavin1* ^−/−^ pup in the cohort died within one day without glucose injections. Right: The red arrow pointed the *Cavin1* ^−/−^ pup, which was rescued by glucose injections and survived at 6 weeks of age.

Before suckling, the blood glucose level in newborn pups is maintained by the mobilization of liver glycogen,^[^ ^14^ ^]^ thus deficits in glucose release from liver glycogen would cause hypoglycemia.^[^ ^15^ ^]^ We assessed the liver glycogen level in *Cavin1* ^−/−^ mice and their wild‐type littermates at 9 h after birth while they are still alive. In wild‐type newborn pups, the fetal liver was full of glycogen before birth and depleted of glycogen within 9 h after birth (**Figure** **2**A,B). However, wild‐type pups maintain healthy blood glucose level (Figure 1F) because the milk intake would catch up with the glucose supply. In contrast, the liver of *Cavin1*‐/‐ fetus contained much less glycogen before birth (Figure 2A,B). Additionally, this stored glycogen could not be efficiently mobilized and 9 h after birth there was still substantial amount of residual glycogen in the liver (Figure 2A,B). Without the glucose supply from liver glycogen the blood glucose level in *Cavin1*‐/‐ pups dropped dramatically and the pups developed lethal hypoglycemia (Figure 1F). The adult *Cavin1*‐/‐ mice that survived after glucose injection could maintain a healthy blood glucose level similar to wild‐type littermates (Figure 2C). However, they still possess abnormal storage and mobilization of liver glycogen (Figure 2D,E). The liver glycogen in wild‐type mice was synthesized and degraded according to the change of food supply, while the liver glycogen in *Cavin1*‐/‐ mice was almost fixed at a low level regardless of the food supply (Figure 2D,E). The liver of homozygous mice could neither store sufficient amount of glycogen during feeding nor efficiently mobilize glycogen during fasting (Figure 2D,E). The liver, skeletal muscle, and heart are the major organs to store glycogen. Glycogen from the liver is degraded and released to regulate blood glucose, while glycogen from skeletal muscle and heart is mostly used by themselves. Cavin1 deficiency only decreased the storage of liver glycogen, but not skeletal muscle or heart glycogen (Figure 2B,E and Figure S2A, Supporting Information). Of these three glycogen storage organs, Cavin1 deficiency only targeted the liver, which could not function as the reservoir for blood glucose regulation. The reduction of glycogen storage and the inhibition of glycogen mobilization indicated that the bilateral flow between liver glycogen and blood glucose was blocked in *Cavin1*‐/‐ mice.

**Figure 2:**
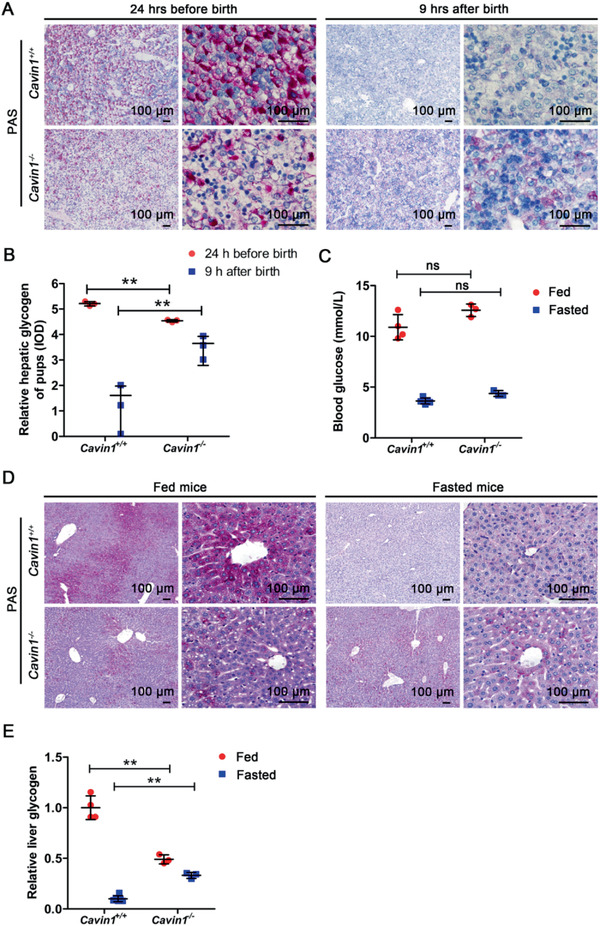
Cavin1 deficiency affected the storage and mobilization of glycogen in the liver. A,B) Periodic acid‐Schiff (PAS) staining and quantitative analysis of hepatic glycogen in wild‐type and Cavin1 deficient newborn pups at 24 h before birth and 9 h after birth. ** *p* < 0.01; *n* = 3 mice each group. C) Blood glucose in adult wild‐type or *Cavin1*‐/‐ homozygous mice when fed or fasted. ns, not significant; *n* = 3–5 mice each group. D,E) PAS staining and quantitative analysis of hepatic glycogen in wild‐type and Cavin1 deficient adult mice when fed and fasted. ** *p* < 0.01; *n* = 3–5 mice each group.

Liver glycogen is synthesized and degraded according to food intake. It is regulated by several cellular signal pathways to balance the activities of glycogen synthase and glycogen phosphorylase. The mitogen‐activated protein (MAP) kinases in the liver are regulated according to the feeding status, i.e., the liver responds to the influx of nutrients following feeding by activating the MAP kinase signal (**Figure** **3**A). However, Cavin1 deficiency specifically inhibited this feeding‐induced MAP kinase in the liver without affecting the same signal cascade in skeletal muscle or heart (Figure 3A). The fixed MAP kinase signal in Cavin1‐deficient mice suggested that some of the metabolic activities in the liver will also be fixed in certain state regardless of the nutritional status. The balance of glycogen synthase and phosphorylase regulates the liver glycogen level. Glycogen phosphorylase is the major enzyme involved in the mobilization of glycogen, catalyzing the reaction of the removal of a glucose residue from the nonreducing end of a glycogen chain.^[^ ^16^ ^]^ We checked the activity of glycogen phosphorylase in the livers of *Cavin1* ^−/−^ and *Cavin1* ^+/+^ mice at 9 h after birth and found that the activity of glycogen phosphorylase was significantly lower in *Cavin1* ^−/−^ livers (Figure 3B), which supported the idea that the absence of Cavin1 would inhibit the glycogen mobilization. Since glycogen mobilization is stimulated mainly by glucagon secreted by pancreatic islets, we considered the possibility that impaired pancreatic function contributed to the deficits in glycogen mobilization in *Cavin1* ^−/−^ pups. Neither *α* nor *β* cells expressed Cavin1 (Figure S3A, Supporting Information). Only CD31‐positive intraislet vascular cells expressed Cavin1 (Figure S3A, Supporting Information). No change in pancreatic islets or islet cells was detected in Cavin1 deficient mice (Figure S3A, Supporting Information). We also assessed the glucagon level in the serum and found that *Cavin1* ^−/−^ mice had a higher level of glucagon than their wild‐type littermates due to their hypoglycemia (Figure 3C). Next, we tested if the Cavin1 deficiency would make the liver not respond to insulin or glucagon signaling. We intraperitoneal injected insulin or glucagon to *Cavin1* ^−/−^ mice and monitored the phosphorylation of AKT and the level of cyclic adenosine monophosphate (cAMP) in the cells. Our data showed that insulin‐induced phosphorylation of AKT level and glucagon‐induced upregulation of cAMP levels were dramatically dampened in *Cavin1* ^−/−^ mice (Figure S3B,C, Supporting Information). These results suggested that the inhibition of glycogen mobilization in the livers is not because of the shortage of glucagon but rather a failure of the glucagon signal reaching hepatic parenchymal cells. Furthermore, to understand the causes of the lower glycogen storage in the liver of *Cavin1* ^−/−^ fetus before birth, we measured the glucose uptake by the liver and the activity of glycogen synthase in the liver, two critical factors for glycogenesis (glycogen synthesis).^[^ ^16^ ^]^ Our data showed that the livers of *Cavin1* ^−/−^ fetus had a significantly lower rate of glucose uptake than the ones of the control fetus (Figure 3D), which is consistent with the lower glycogen storage in their livers before birth. In contrast, the livers of *Cavin1* ^−/−^ fetus had a slightly higher activity of glycogen synthase than the ones of the control fetus (Figure 3E). We also observed a higher level of phosphorylated glycogen synthase kinase 3*β* (GSK3*β*) at Ser9 and a lower level of phosphorylated glycogen synthase at Ser641 (Figure 3F), and both findings were in line with higher glycogen synthase activity (Figure 3E). One explanation for this inconsistency is that the higher activity of glycogen synthase in the liver of *Cavin1* ^−/−^ fetus could be a mechanism for the liver to compensate for the shortage of glycogen due to the limited glucose uptake. The glucose in blood crosses the sinusoidal endothelial wall and enters the disse space where it is uptaken by hepatocytes with the help of glucose transporters.^[^ ^17^, ^18^ ^]^ Western blot analysis revealed no change in the expression and distribution of glucose transporters (Glut1 and Glut2) in the liver of Cavin1‐deficient mice (Figure 3G,H). In order to rule out the possibility that any defects in hepatocyte caused the abnormal glycogen metabolism, we isolated primary hepatocytes from Cavin1‐deficient mice and treated with insulin and glucagon. We found that the hepatocytes from *Cavin1* ^−/−^ mice had no problem to store or mobilize glycogen upon the treatment of insulin or glycagon (Figure 3I,J). Thus, the lower rate of glucose uptake in Cavin1 null mice is because of insufficient amount of glucose in disse space, rather than the inability of the hepatocytes to transport glucose. In summary, our data demonstrated that the Cavin1 deficiency blocked the bilateral flow between liver glycogen and blood glucose and this led to abnormal storage and mobilization of glycogen in mouse liver and the eventual hypoglycemia in *Cavin1* ^−/−^ mice.

**Figure 3:**
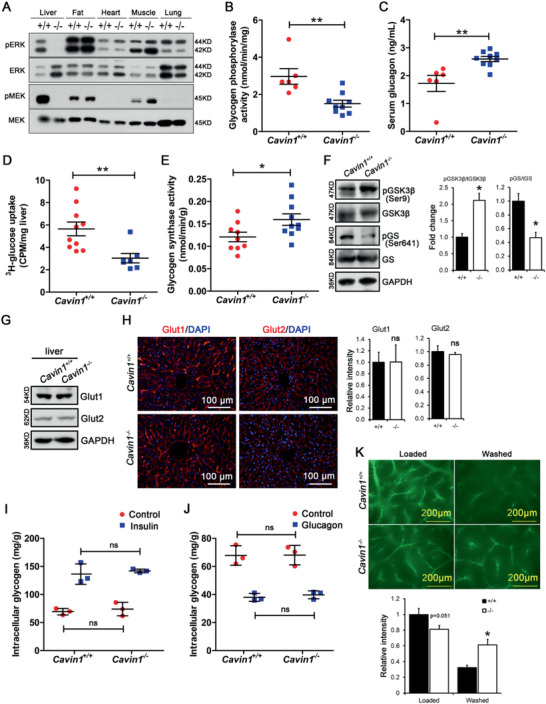
Cavin1 deficiency impaired the metabolism of liver glycogen. A) Specific inhibition of MAP kinase signal in liver by Cavin1 deficiency. Tissues (*liver*, *fat*, *heart*, *muscle*, and *lung*) of normally fed wild‐type (*+/+*) and *Cavin1^‐/‐^* mice (*‐/‐*) were obtained for protein analysis. Phosphorylated extracellular signal‐ regulated kinase (ERK) and mitogen‐activated protein kinase kinase (MEK) and total ERK and MEK were analyzed by western blot. B,C) Glycogen phosphorylase activity in liver and serum glucagon level in *Cavin1* ^+/+^ and *Cavin1* ^−/−^ pups at 9 h after birth. ** *p *< 0.01; *n* = 6–9 mice each group. D) Quantitative analysis of ^3^H‐labeled glucose uptake to liver in *Cavin1* ^+/+^ and *Cavin1* ^−/−^ pups. ** *p* < 0.01; *n* = 7–10 mice each group. E) Measurement of glycogen synthase activity in *Cavin1* ^+/+^ and *Cavin1* ^−/−^ fetus. ** *p* < 0.01; *n* = 9–10 mice each group. F) Western blot analysis for related glycogen synthesis enzymes including glycogen synthase (GS), phosphorylated glycogen synthase (pGS(S641)), glycogen synthase kinase 3*β* (GSK3*β*), and its inactive form (pGSK3*β*(Ser9)) in *Cavin1* ^+/+^ and *Cavin1* ^−/−^ fetus. glyceraldehyde‐3‐phosphate dehydrogenase (GAPDH) serves as loading control. The intensity of the bands were quantified and normalized with GAPDH. The ratio between pGSK3*β* and GSK3*β* in *Cavin1* ^+/+^ (marked as +/+) and *Cavin1* ^−/−^ (‐/‐) mice were calculated. The fold change between two groups was plotted. G) The expression and H) distribution of Glut1 and Glut2 in the livers of *Cavin1* ^+/+^ and *Cavin1* ^−/−^ fetus. I) The glycogen stored in the primary hepatocytes isolated from *Cavin1* ^+/+^ and *Cavin1* ^−/−^ mice upon insulin treatment. ns, not significant. J) The leftover glycogen in the primary hepatocytes isolated from *Cavin1* ^+/+^ and *Cavin1* ^−/−^ mice upon glucagon treatment. ns, not significant. K) fluorescein isothiocyanate (FITC)‐dextran were perfused in the livers of *Cavin1* ^+/+^ (+/+) and *Cavin1* ^−/−^ (‐/‐) mice. FITC‐dextran crossed the endothelial walls and accumulated in the Disse's place. The pictures of “Loaded” liver were taken and the intensity of the fluorescence was quantified and the relative intensity was plotted. After 20 min washing, the pictures were taken on these “Washed” livers. These pictures were quantified and analyzed similar to the loaded livers. * *p* < 0.05; *n* = 3 mice each group.

In order to understand how Cavin1 deficiency impacts liver function, we investigated in which cell populations the *Cavin1* expresses. Immunostaining in liver samples revealed that Cavin1 predominantly expressed in the endothelial cells of sinusoids, portal venule, and central veins (**Figure** **4**A and Figure S4A,B, Supporting Information). A recent publication provided single‐cell RNA sequencing data on nonparenchymal cells isolated from the livers of wild‐type C57BL/6J mice.^[^ ^19^ ^]^ We reanalyzed these data and clustered all cells by the Louvain algorithm. We then performed dimensionality reduction for presentation using t‐distributed stochastic neighbor embedding and uniform manifold approximation and projection (Figure 4B). All clusters were marked by known markers of different cell populations existing in the liver. Our data showed that *Cavin1* only expressed in cluster 0 and cluster 9, which were marked as endothelial cells and hepatic stellate cells, respectively (Figure 4C). These results implied that the loss of Cavin1 might affect the endothelial cells or hepatic stellate cells instead of hepatic parenchymal cells. Thus, we evaluated hepatic tissue morphology (Figure 4D), the expression of endothelial markers (CD36, VEGFR2, and Tie2) (Figure 4E,F), and calculated the vascular network distribution (Figure 4G). Taken together, our results showed that Cavin1 predominantly expressed in the endothelial cells, but there is no differences in the vascular network between wild‐type and Cavin1‐deficient mice (Figure 4A–G).

**Figure 4:**
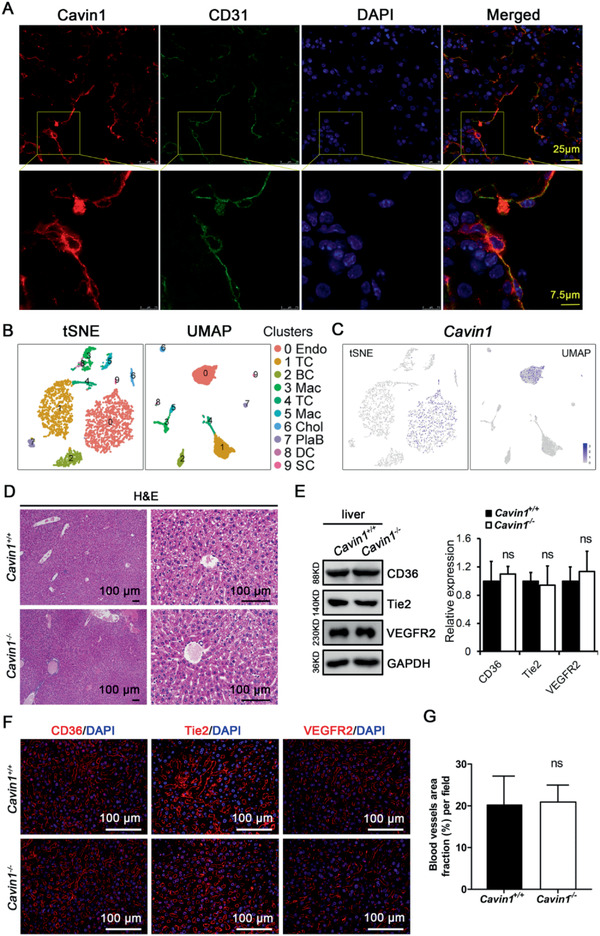
Cavin1 deficiency did not affect liver vascular network. A) The expression of Cavin1 in endothelial cells but not in hepatocytes. Liver of wild‐type mouse was stained for Cavin1 and CD31. B) Nonparenchymal cells from the livers were clustered into ten populations and each population was marked by known cell marker. Endo: Endothelial cells; TC: T cells; BC: B cells; Mac: macrophages; Chol: cholangiocyte; PlaB: plasma B cell; DC: dendritic cells, and SC: hepatic stellate cells. C) Cavin1 labeled cells were in cluster 0 and cluster 9, identified as endothelial cells and hepatic stellate cells, respectively. D) Hematoxylin‐eosin staining to reveal the central veins/portal venule and sinusoids in *Cavin1* ^+/+^ and *Cavin1* ^−/−^ liver. E,F) The expression and distribution of vascular markers: CD36, Tie2, and VEGFR2 in *Cavin1* ^+/+^ and *Cavin1* ^−/−^ liver. GAPDH served as loading control. G) Quantitative analysis of blood vessels area fraction in *Cavin1* ^+/+^ and *Cavin1* ^−/−^ liver. ns, not significant; *n* = 3 mice each group.

The permeability of the sinusoidal endothelial wall determined the amount of glucose, lipids, proteins, and other molecules brought in and out of the liver.^[^ ^20^ ^]^ The unchanged vascular network suggested that the permeability of the sinusoidal endothelial wall might be affected by Cavin1 deficiency. It was believed that the permeability of the sinusoidal endothelial wall is regulated by different endothelial subcellular structures: caveolae, fenestrae, and transendothelial channels (TECs).^[^ ^21^ ^]^ There is little knowledge of the function of TECs, which are thought to be fenestrae precursors.^[^ ^22^ ^]^ Thus, we checked if the deficiency of Cavin1 impaired the endothelial permeability and caused the disorder of glycogen metabolism by affecting the formation of caveolae and/or fenestrae. Caveolae have two essential component proteins, Caveolin1 and Cavin1, and both proteins are equally necessary for caveolae formation. Lacking either one ablates caveolae completely.^[^ ^8^, ^23^ ^]^ We backcrossed *Cav1* ^+/−^ mice over 20 generations to pure C57BL/6J background and then crossed *Cav1* ^+/−^ to generate Caveolin1 null mice. Surprisingly, no neonatal death occurred in *Cav1* ^−/−^ newborn pups, suggesting that ablation of caveolae did not mimic the phenotype in Cavin1 null mice (**Figure** **5**A). We also bred some *Cavin1* ^+/−^::*Cav1* ^−/−^ to generate *Cavin1* ^−/−^::*Cav1* ^−/−^ double knockout mice. These double knockout died at the neonatal stage, similar to the *Cavin1* ^−/−^ mice (Figure 5A). The ablation of caveolae in *Cav1* ^−/−^ mice did not alter the liver glycogen as in the *Cavin1* ^−/−^ mice (Figure 5B). These results indicated that the deficiency of Cavin1 caused neonatal death through a caveolae‐independent mechanism. Although the depletion of caveolin1 dramatically decreased the expression level of Cavin1 and other members of the Cavins family in mouse liver (Figure 5C), it seems that the residual Cavin1 is enough for the survival of these *Cav1* knockout mice.

**Figure 5:**
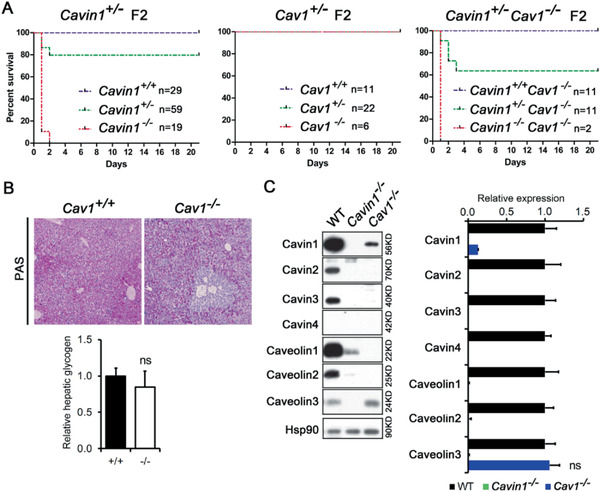
The neonatal death of Cavin1 null mice is independent of Caveolae or Caveolin1. A) The survival rate of newborn pups of *Cavin1* ^+/−^, *Cav1* ^+/−^, *Cav1* ^+/−^::*Cavin1* ^−/−^ heterozygous parents. The numbers of pups in each group were listed in the figure. B) Periodic acid‐Schiff staining for liver glycogen. Liver from normally fed adult wild‐type or Caveolin1 deficient mice was stained for glycogen. Hepatic glycogen was calculated by the PAS positive area and relative hepatic glycogen was plotted. ns, not significant; *n* = 3 mice each group. C) Western blot analysis of Cavin1–4 and Caveolin1‐3 expressed in Cavin1 or Caveolin1 deficient mice. Hsp90 served as loading control. The relative expression level of each protein was calculated based on western blot results. ns, not significant. All other comparisons between *Cavin1* ^−/−^ and wild‐type (WT) or between *Cav1* ^+/−^ and WT groups reach significant.

Next, we tested if Cavin1 deficiency impaired endothelial permeability and glycogen metabolism by disrupting fenestrae formation. It has been reported that prominently increased number of fenestration in liver sinusoidal endothelial cells (LSECs) can promote glucose uptake in liver,^[^ ^24^ ^]^ while a decreased number of fenestration of LSECs can significantly impair the absorption of glucose in the liver, thereby affecting hepatic glycogen synthesis.^[^ ^25^, ^26^ ^]^ First, we compared the morphology of fenestrae in the liver of wild‐type and *Cavin1* ^−/−^ mice by transmission electron microscope (TEM) and scanning electron microscope (SEM). Our results confirmed that the density of fenestrae in the liver of *Cavin1* ^−/−^ mice was less than in wild‐type mice (**Figure** **6**A). The results from SEM examination on primary cultured LSECs also demonstrated that primary LSECs isolated from *Cavin1* ^−/−^ mice contained fewer fenestrae, compared with that from wild‐type mice (Figure 6B). Quantitatively, in Cavin1‐deficient mice, the number of fenestrae in LSECs was reduced by half (Figure 6C). Combined, our data verified that the depletion of Cavin1 resulted in a decreased amount of fenestrae in the LSECs. Next, we tested if the reduction of fenestrae in LSECs would lead to glycogen disorder, mimicking the effect of Cavin1 deficiency. Here we used two known fenestrae inhibitors, histamine and nicotine^[^ ^27^, ^28^ ^]^ to treat adult wild‐type C57BL/6J mice and measured how much residual glycogen was left after fasting for 6 h. Our results showed that the mice treated with the inhibitors had more leftover glycogen in their liver (Figure 6D,E), suggesting that the reduction of the fenestrae decreased the mobilization of liver glycogen to the bloodstream. Reversely, we found that histamine and nicotine also blocked glycogen formation during fed state (Figure 6F). In summary, our results indicated that the loss of Cavin1 resulted in fewer fenestrae in LSECs and consequently blocked bilateral flow between liver glycogen and blood glucose.

**Figure 6:**
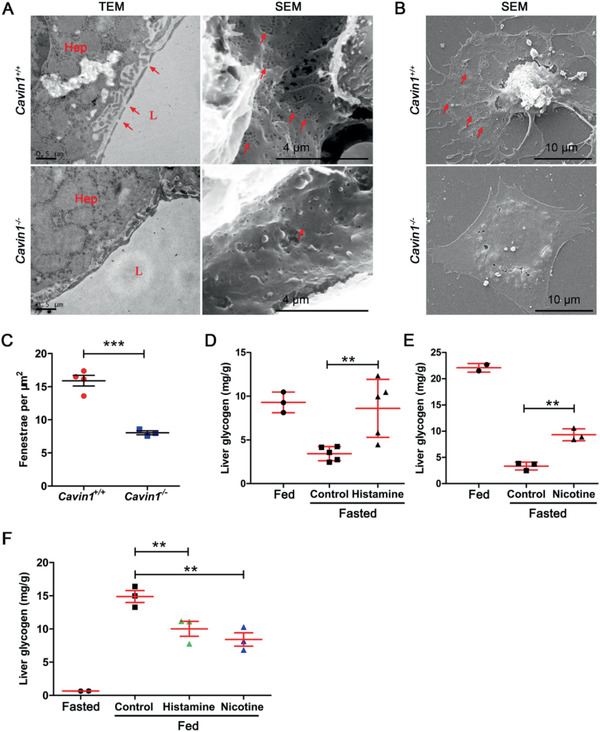
Cavin1 deficiency dampened the fenestration in LSECs. A) Transmission (TEM) and scanning electron microscopy (SEM) showed fenestrae in the livers. Red arrows indicated the fenestrae; Hep, hepatocyte; L, lumen of the sinusoid. B) SEM showed fenestrae in primary cultured LSECs from *Cavin1* ^+/+^ and *Cavin1* ^−/−^ mice. C) Quantitative analysis of fenestrae in the LSECs. *** *p* < 0.001; each dot stands for one SEM picture of LSECs; LSECs are from three to five mice each group. D,E) The hepatic glycogen content in adult wild‐type mice at fed and 6 h after fasting. Treatments with D) histamine or E) nicotine inhibited glycogen mobilization upon fasting. ** *p* < 0.01; *n* = 2–5 mice each group. F) The hepatic glycogen content in adult wild‐type mice after 6 h refeeding. Treatments with histamine or nicotine inhibited glycogen storage upon refeeding.

The fenestrae in LSECs are formed after cell differentiation.^[^ ^17^, ^18^ ^]^ Initially, the lining of a liver sinusoid is a continuous endothelial wall with an outer basement membrane. After differentiation the endothelial lining becomes fenestrated and the outside basement membrane disappears. The marker protein of CD31 expressed in undifferentiated LSECs also becomes the differentiated cell expressed CD4.^[^ ^17^, ^18^ ^]^ Cavin1 was expressed in LSECs since early developmental stage of LSECs (Figure S5A, Supporting Information). Cavin1 deficiency did not alter the endothelial nature of LSECs before birth (**Figure** **7**A) or at weaning (Figure 7B). What was affected by Cavin1 deficiency was the differentiation of LSECs. In Cavin1‐deficient mice the expression of undifferentiated LSEC marker CD31 was high and the expression of differentiated marker CD4 was low (Figure 7C). There were also more basement membrane proteins, laminin *β*1 (LAMB1) and collagen *α*1 (IV) (COL4A1) (Figure 7D). The reduced fenestration plus incomplete loss of outside basement membrane indicated that the impairment of LSEC differentiation was due to the absence of Cavin1.

**Figure 7:**
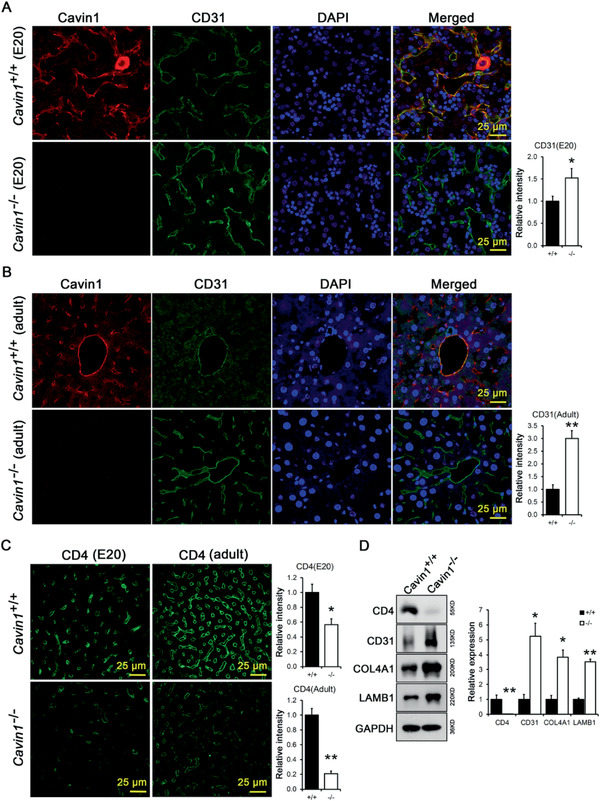
Incomplete differentiation of LSEC in Cavin1 deficient mice. A,B) Increased CD31 expression in LSECs of Cavin1 deficient mice. Liver of E20 fetus or adult mice (8 weeks) was stained for Cavin1 and CD31. * *p* < 0.05; ** *p* < 0.01; *n* = 3–5 mice per group. C) Inhibited CD4 expression in LSECs of Cavin1 deficient mice. * *p* < 0.05; ** *p* < 0.01; *n* = 3–5 mice per group. D) Analysis of endothelial marker proteins and basement membrane proteins in liver of *Cavin1* ^+/+^ and *Cavin1* ^−/−^ mice. COL4A1, collagen *α*1(IV); LAMB1, laminin *β*1. GAPDH served as loading control. All immuno‐fluorescence and western blot picture were analyzed and the relative intensity from each group were plotted on the bar graph.

Fenestrae are sensitive to environmental conditions and change in number and diameter in response to external stimuli such as hormones, drugs, and toxins.^[^ ^27^, ^29^, ^30^ ^]^ The molecular mechanisms regulating their structure are not fully understood, but previous studies have shown that the Rho signaling pathway regulates fenestrae formation by promoting the dynamics of the actin cytoskeleton.^[^ ^31^, ^32^, ^33^, ^34^ ^]^ Active Rho (guanosine triphosphate (GTP)–bound Rho) regulates Cofilin via Rho‐associated protein kinase (ROCK) and LIM domain kinase (LIMK).^[^ ^35^, ^36^ ^]^ Cofilin is an actin‐binding protein and is essential for the rapid turnover of the actin filament.^[^ ^36^ ^]^ By inducing Cofilin phosphorylation, Rho abolishes the actin‐binding activity of Cofilin, thereby enhancing the dynamics of actin filaments.^[^ ^37^ ^]^ In addition, it has been reported that Cavin4/MURC, one of the Cavin1 homologs, can activate RhoA‐ROCK in cardiomyocytes.^[^ ^38^ ^]^ To test whether the knockout of *Cavin1* affected the fenestrae through the RhoA‐ROCK signal pathway in LSECs, we investigated the level of active RhoA and its downstream ROCK2‐LIMK‐Cofilin signaling pathway in our newborn wild‐type and *Cavin1* knockout mice. We used anti‐RhoA‐GTP antibodies to immunoprecipitate the active RhoA (RhoA‐GTP) and found that the level of active RhoA was significantly lower in the liver of *Cavin1* ^−/−^ mice when compared to wild‐type mice (**Figure** **8**A). We also assessed the amount of ROCK2 associated with active RhoA by immuno‐blotting the anti‐RhoA‐GTP co‐immunoprecipitated mixture with the ROCK2 antibody. Our data showed that the level of ROCK2 associated with active RhoA was lower in *Cavin1* ^−/−^ mice (Figure 8A). Furthermore, our analysis revealed that the phosphorylated level of LIMK1 at Thr508 and Cofilin at Ser3 were also decreased in the liver of Cavin1‐deficient mice, which is consistent with the lower activity of RhoA (Figure 8B). Subsequently, we evaluated the level of actin associated with Cofilin by co‐immunoprecipitation with Cofilin antibody. Our results showed that the level of actin associated with Cofilin was increased in the liver of *Cavin1* ^−/−^ mice, suggesting fewer dynamics of actin cytoskeleton than that in *Cavin1* ^+/+^ mice (Figure 8C). In order to prove that the observed inhibition of RhoA‐ROCK2‐LIMK‐Cofilin signaling pathway is not coming from other liver cell types, we performed similar experiments in an endothelial cell line, human umbilical vein endothelial cells (HUVEC). Using siRNAs, we successfully knocked down the expression of Cavin1 in HUVECs (Figure S6A, Supporting Information) and confirmed that depletion of Cavin1 inhibited RhoA‐ROCK2‐LIMK‐Cofilin signaling pathway in HUVECs as well (Figure S6B–D, Supporting Information). Combined, our data demonstrated that the LSECs from *Cavin1* ^−/−^ mice had inhibited RhoA‐ROCK2‐LIMK‐Cofilin signaling pathway and impaired dynamics of cytoskeleton, which would eventually result in a reduction of fenestrae. Furthermore, it had been reported that latrunculin A could promote the dynamic cytoskeleton remodeling and lead to the formation of fenestrae in the LSECs.^[^ ^39^ ^]^ Thus, we tested if the treatment with latrunculin A would be able to rescue the reduction of fenestrae in the LSECs caused by Cavin1 deficiency. Indeed, the treatment of latrunculin A caused massive depolymerization of F‐actin (Figure 8D) and induced substantial fenestration in the primary cultured LSECs from *Cavin1* ^−/−^ mice (Figure 8E). We then went further to test if latrunculin A treatment would rescue *Cavin1* ^−/−^ mice from neonatal death. Indeed, two injections of 1.25 mg kg^−1^ of latrunculin A, at 1 and 5 h after birth, rescued *Cavin1* ^−/−^ mice (Figure 8F) and the rescued mice presented a healthy blood glucose level at 9 h after birth (Figure 8G). Collectively, our data suggested that the loss of Cavin1 impaired the dynamics of the actin cytoskeleton through downregulation of RhoA signaling pathway and resulted in a reduction of fenestrae in the LSECs.

**Figure 8:**
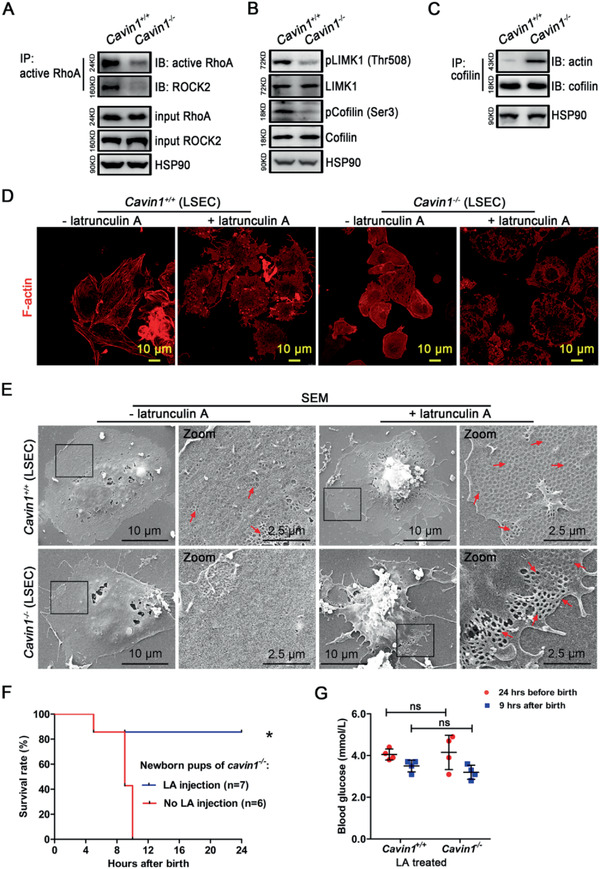
Cavin1 deficiency inhibited the dynamics of the actin cytoskeleton and reduced the fenestrae in LSECs. A) Western blot analysis of active Rho A and its binding with ROCK2 in the liver of *Cavin1* ^+/+^ and *Cavin1* ^−/−^ pups. Input Rho A, input ROCK2 and HSP90 are shown as control. B) Western blot analysis of phosphorylated LIMK1 at Thr508 and phosphorylated cofilin at Ser3 in the liver lysate of *Cavin1* ^+/+^ and *Cavin1* ^−/−^ pups. C) Western blot analysis of cofilin co‐immunoprecipitation with actin in the liver lysate of *Cavin1* ^+/+^ and *Cavin1* ^−/−^ pups. D) Immunofluorescent staining of F‐actin in LSECs treated with or without latrunculin A (1 µg mL^−1^). E) Scanning electron microscopy analysis of fenestrae in the LSECs with or without latrunculin A treatment. Red arrows indicate fenestrae in the LSECs. Zoom shows the details of the enlarged area (black squares). F) The survival rate of *Cavin1* ^−/−^ pups when treated with Latrunculin A (LA). * *p* < 0.05; *n* = 6–7 mice per group. G) The blood glucose in LA treated mice. ns, not significant; *n* = 4 mice each group.

In summary, our data demonstrated that the depletion of Cavin1 impaired the permeability of hepatic sinusoidal capillaries by decreasing the number of fenestrae in LSECs and affected the homeostasis of glycogen, which led to the neonatal death of *Cavin1* ^−/−^ mice in C57BL/6J background. So far, there is no recorded neonatal death of CGL4 patients, but this may be because CGL4 patients are rare and often living in rural areas. One clinical report mentioned that a sister of their CGL4 patient died 6 h after birth,^[^ ^40^ ^]^ but her genotype and the cause of death were not recorded. Thus, it is not clear if her death was caused by a *CAVIN1* mutation. Nevertheless, our results raised the concern that Cavin1 deficiency may cause neonatal mortality in CGL4 patients. It is worth pointing out that the neonatal death of Cavin1 null mice only occurs in certain genetic background, such as C57BL/6J. Once we cross these mice with Friend Virus B (FVB), the *Cavin1* ^−/−^ mice with 50% FVB background can improve survival with mild neonatal hypoglycemia (Figure S7, Supporting Information). This points to the impact of genetic background on susceptibility to the disorder of glucose homeostasis.^[^ ^41^, ^42^, ^43^ ^]^ A more recent study on PV1, an essential protein for endothelial permeability, also showed that the deletion of PV1 is 100% lethal in pure C57BL/6J background but causes a much milder defect in ones with other backgrounds.^[^ ^22^ ^]^ Given the complexity of human genetic background, neonatal death may only occur in few CGL4 patients with a certain background. Nevertheless, our finding suggests close monitoring on glucose level for newborn CGL4 patients.

The roles of fenestration and permeability of LSECs in liver diseases have drawn more attention in recent years.^[^ ^22^, ^44^ ^]^ The disorder of substance exchange between blood and hepatic parenchyma leads to the development of liver‐related metabolic diseases.^[^ ^17^ ^]^ In fact, some previously reported phenotypes of *Cavin1* ^−/−^ mice, such as glucose intolerance and hyperinsulinemia, are metabolically related to liver functions.^[^ ^45^ ^]^ The novel function of Cavin1 in regulating the fenestration of LSECs may partially contribute to the phenotypes in *Cavin1* knockout mice. A mouse model with an endothelial specific knockout of Cavin1 is needed for further study on the roles of endothelial Cavin1 in liver diseases.

Fenestrae are dynamic structures. Their numbers and diameters are affected by physiological and pathological factors such as aging, nutritional status (feeding or fasting) or diseases (cirrhosis, stress‐related disorders, cancer, etc.),^[^ ^29^, ^32^, ^46^ ^]^ but the molecular mechanisms regulating their structure remain largely elusive. A decade ago, LSECs fenestrations were thought to be sort of caveolae.^[^ ^47^ ^]^ However, Caveolin1 null mice have normal fenestrations,^[^ ^48^ ^]^ suggesting that normal structure of fenestrations in the LSECs is not dependent upon Caveolin1 or caveolae. Indeed, our results showed that neonatal Caveolin1 null mice had normal survival rates (Figure 5A), while both Cavin1 single knockout and Cavin1/Cav1 double knockout resulted in neonatal death. Taken together, we ruled out the possibility that Cavin1 regulates the fenestration of LSECs in a caveolae‐dependent manner. Instead, our data suggested that the loss of Cavin1 inhibited the differentiation of LSECs (Figure 7A–D) and impaired the dynamic of actin cytoskeleton through the downregulation of the RhoA signaling pathway (Figure 8A–E). The activity of RhoA is regulated by three kinds of regulators: guanine nucleotide exchange factors, GTPase‐activating proteins, and Rho guanosine diphosphate dissociation inhibitors. Cavin1 has no typical motif as any of these three regulators. Thus, Cavin1 probably interacts with molecules that directly or indirectly regulate the activity of upstream regulators for RhoA, which subsequently promotes the dynamics of the actin skeleton. Our immunostaining results showed that Cavin1 is not colocalizing with F‐actin (Figure S8A, Supporting Information). Instead, they are locating adjacent to or right in the empty rings of the F‐actin (Figure S8A, Supporting Information), suggesting that the Cavin1 may cause the depolymerization of F‐actin through an effector locally. One of the future directions of this study will be to find the effector of Cavin1 and we will test if Cavin1‐associated proteins are such potential targets. The analysis on single‐cell RNA sequencing data suggested that Cavin1 also expresses in hepatic stellate cells. How does the Cavin1 deficiency in hepatic stellate cells contribute to the abnormalities of *Cavin1* ^−/−^ mice will be an interesting question to ask. It is also intriguing to know how Cavin1 is regulated according to extracellular stimuli. A recent study showed that, upon insulin stimulation, Cavin1 is acutely translocated to focal complex compartments and regulates focal complex formation in adipocytes.^[^ ^29^ ^]^ Whether similar mechanism also exists in LSECs will need further studies.

## Experimental Section

Materials and methods are described in detail in the Supporting Information. All experiments were performed with approval under the ethical guidelines of the Institute of Biochemistry and Cell Biology.

## Conflict of Interest

The authors declare no conflict of interest.

## Supporting information

Supporting Information

## References

[1] N. Patni , A. Garg , Nat. Rev. Endocrinol. 2015, 11, 522. 26239609 10.1038/nrendo.2015.123 PMC7605893

[2] G. Nilay , T. Kutlu , G. T. Tekant , A. G. Eroglu , N. C. Ustundag , B. Ozturk , H. Onay , B. Tuysuz , Eur. J. Med. Genet. 2020, 63, 103819. 31778856 10.1016/j.ejmg.2019.103819

[3] M. M. Hill , M. Bastiani , R. Luetterforst , M. Kirkham , A. Kirkham , S. J. Nixon , P. Walser , D. Abankwa , V. M. Oorschot , S. Martin , J. F. Hancock , R. G. Parton , Cell 2008, 132, 113. 18191225 10.1016/j.cell.2007.11.042 PMC2265257

[4] L. Liu , Biochem. Soc. Trans. 2020, 48, 147. 31922193 10.1042/BST20190380 PMC7080641

[5] N. Briand , I. Dugail , S. Le Lay , Biochimie 2011, 93, 71. 20363285 10.1016/j.biochi.2010.03.022

[6] R. G. Parton , M. A. del Pozo , Nat. Rev. Mol. Cell Biol. 2013, 14, 98. 23340574 10.1038/nrm3512

[7] R. Gupta , C. Toufaily , B. Annabi , Biochimie 2014, 107, 188. 25241255 10.1016/j.biochi.2014.09.010

[8] L. Liu , D. Brown , M. McKee , N. K. Lebrasseur , D. Yang , K. H. Albrecht , K. Ravid , P. F. Pilch , Cell Metab. 2008, 8, 310. 18840361 10.1016/j.cmet.2008.07.008 PMC2581738

[9] M. S. Karbalaei , C. Rippe , S. Albinsson , M. Ekman , A. Mansten , B. Uvelius , K. Sward , Eur. J. Pharmacol. 2012, 689, 179. 22643325 10.1016/j.ejphar.2012.05.023

[10] C. G. Hansen , E. Shvets , G. Howard , K. Riento , B. J. Nichols , Nat. Commun. 2013, 4, 1831. 23652019 10.1038/ncomms2808 PMC3674239

[11] T. Taniguchi , N. Maruyama , T. Ogata , T. Kasahara , N. Nakanishi , K. Miyagawa , D. Naito , T. Hamaoka , M. Nishi , S. Matoba , T. Ueyama , PLoS One 2016, 11, e0162513. 27612189 10.1371/journal.pone.0162513 PMC5017623

[12] M. H. Lees , J. Pediatr. 1970, 77, 484. 5502102 10.1016/s0022-3476(70)80024-5

[13] B. Turgeon , S. Meloche , Physiol. Rev. 2009, 89, 1. 19126753 10.1152/physrev.00040.2007

[14] J. Girard , P. Ferre , J. P. Pegorier , P. H. Duee , Physiol. Rev. 1992, 72, 507. 1557431 10.1152/physrev.1992.72.2.507

[15] N. D. Wang , M. J. Finegold , A. Bradley , C. N. Ou , S. V. Abdelsayed , M. D. Wilde , L. R. Taylor , D. R. Wilson , G. J. Darlington , Science 1995, 269, 1108. 7652557 10.1126/science.7652557

[16] H. S. Han , G. Kang , J. S. Kim , B. H. Choi , S. H. Koo , Exp. Mol. Med. 2016, 48, e218. 26964834 10.1038/emm.2015.122 PMC4892876

[17] J. Poisson , S. Lemoinne , C. Boulanger , F. Durand , R. Moreau , D. Valla , P. E. Rautou , J. Hepatol. 2017, 66, 212. 27423426 10.1016/j.jhep.2016.07.009

[18] K. K. Sorensen , J. Simon‐Santamaria , R. S. McCuskey , B. Smedsrod , Compr. Physiol. 2015, 5, 1751. 26426467 10.1002/cphy.c140078

[19] X. Xiong , H. Kuang , S. Ansari , T. Liu , J. Gong , S. Wang , X. Y. Zhao , Y. Ji , C. Li , L. Guo , L. Zhou , Z. Chen , P. Leon‐Mimila , M. T. Chung , K. Kurabayashi , J. Opp , F. Campos‐Perez , H. Villamil‐Ramirez , S. Canizales‐Quinteros , R. Lyons , C. N. Lumeng , B. Zhou , L. Qi , A. Huertas‐Vazquez , A. J. Lusis , X. Z. S. Xu , S. Li , Y. Yu , J. Z. Li , J. D. Lin , Mol. Cell 2019, 75, 644. 31398325 10.1016/j.molcel.2019.07.028 PMC7262680

[20] K. Auvinen , E. Lokka , E. Mokkala , N. Jappinen , S. Tyystjarvi , H. Saine , M. Peurla , S. Shetty , K. Elima , P. Rantakari , M. Salmi , Sci. Rep. 2019, 9, 15698. 31666588 10.1038/s41598-019-52068-x PMC6821839

[21] E. Tkachenko , D. Tse , O. Sideleva , S. J. Deharvengt , M. R. Luciano , Y. Xu , C. L. McGarry , J. Chidlow , P. F. Pilch , W. C. Sessa , D. K. Toomre , R. V. Stan , PLoS One 2012, 7, e32655. 22403691 10.1371/journal.pone.0032655 PMC3293851

[22] R. V. Stan , D. Tse , S. J. Deharvengt , N. C. Smits , Y. Xu , M. R. Luciano , C. L. McGarry , M. Buitendijk , K. V. Nemani , R. Elgueta , T. Kobayashi , S. L. Shipman , K. L. Moodie , C. P. Daghlian , P. A. Ernst , H. K. Lee , A. A. Suriawinata , A. R. Schned , D. S. Longnecker , S. N. Fiering , R. J. Noelle , B. Gimi , N. W. Shworak , C. Carriere , Dev. Cell 2012, 23, 1203. 23237953 10.1016/j.devcel.2012.11.003 PMC3525343

[23] M. Drab , P. Verkade , M. Elger , M. Kasper , M. Lohn , B. Lauterbach , J. Menne , C. Lindschau , F. Mende , F. C. Luft , A. Schedl , H. Haller , T. V. Kurzchalia , Science 2001, 293, 2449. 11498544 10.1126/science.1062688

[24] M. Mohamad , S. J. Mitchell , L. E. Wu , M. Y. White , S. J. Cordwell , J. Mach , S. M. Solon‐Biet , D. Boyer , D. Nines , A. Das , S. Y. Catherine Li , A. Warren , S. N. Hilmer , R. Fraser , D. A. Sinclair , S. J. Simpson , R. de Cabo , D. G. Le Couteur , V. C. Cogger , Aging Cell 2016, 15, 706. 27095270 10.1111/acel.12481 PMC4933657

[25] S. M. Raines , O. C. Richards , L. R. Schneider , K. L. Schueler , M. E. Rabaglia , A. T. Oler , D. S. Stapleton , G. Genove , J. A. Dawson , C. Betsholtz , A. D. Attie , Am. J. Physiol.: Endocrinol. Metab. 2011, 301, E517. 21673305 10.1152/ajpendo.00241.2011 PMC3174531

[26] L. Herrnberger , R. Hennig , W. Kremer , C. Hellerbrand , A. Goepferich , H. R. Kalbitzer , E. R. Tamm , PLoS One 2014, 9, e115005. 25541982 10.1371/journal.pone.0115005 PMC4277272

[27] F. Braet , E. Wisse , Comp. Hepatol. 2002, 1, 1. 12437787 10.1186/1476-5926-1-1 PMC131011

[28] J. N. O'Reilly , V. C. Cogger , D. G. Le Couteur , J. Electron Microsc. 2010, 59, 65. 10.1093/jmicro/dfp039 19648606

[29] F. Braet , Liver Int. 2004, 24, 532. 15566501 10.1111/j.1478-3231.2004.0974.x

[30] F. Braet , M. Shleper , M. Paizi , S. Brodsky , N. Kopeiko , N. Resnick , G. Spira , Comp. Hepatol. 2004, 3, 7. 15341660 10.1186/1476-5926-3-7 PMC519024

[31] H. Yokomori , K. Yoshimura , S. Funakoshi , T. Nagai , K. Fujimaki , M. Nomura , H. Ishii , M. Oda , Lab. Invest. 2004, 84, 857. 15107805 10.1038/labinvest.3700114

[32] H. Yokomori , Med. Mol. Morphol. 2008, 41, 1. 18470674 10.1007/s00795-007-0390-7

[33] H. Yokomori , K. Yoshimura , T. Nagai , K. Fujimaki , M. Nomura , T. Hibi , H. Ishii , M. Oda , Hepatol. Res. 2004, 30, 169. 15588783 10.1016/j.hepres.2004.08.002

[34] C. Xu , X. Wu , B. K. Hack , L. Bao , P. N. Cunningham , Physiol. Rep. 2015, 3. 10.14814/phy2.12636 PMC4760430 26634902

[35] S. T. Sit , E. Manser , J. Cell Sci. 2011, 124, 679. 21321325 10.1242/jcs.064964

[36] P. Lappalainen , D. G. Drubin , Nature 1997, 388, 78. 9214506 10.1038/40418

[37] J. R. Bamburg , A. McGough , S. Ono , Trends Cell Biol. 1999, 9, 364. 10461190 10.1016/s0962-8924(99)01619-0

[38] T. Ogata , T. Ueyama , K. Isodono , M. Tagawa , N. Takehara , T. Kawashima , K. Harada , T. Takahashi , T. Shioi , H. Matsubara , H. Oh , Mol. Cell. Biol. 2008, 28, 3424. 18332105 10.1128/MCB.02186-07 PMC2423172

[39] F. Braet , R. De Zanger , D. Jans , I. Spector , E. Wisse , Hepatology 1996, 24, 627. 8781335 10.1053/jhep.1996.v24.pm0008781335

[40] A. Ardissone , C. Bragato , L. Caffi , F. Blasevich , S. Maestrini , M. L. Bianchi , L. Morandi , I. Moroni , M. Mora , BMC Med. Genet. 2013, 14, 89. 24024685 10.1186/1471-2350-14-89 PMC3846852

[41] E. H. Leiter , D. L. Coleman , K. P. Hummel , Diabetes 1981, 30, 1029. 7030828 10.2337/diab.30.12.1029

[42] S. Kooptiwut , S. Zraika , A. W. Thorburn , M. E. Dunlop , R. Darwiche , T. W. Kay , J. Proietto , S. Andrikopoulos , Endocrinology 2002, 143, 2085. 12021173 10.1210/endo.143.6.8859

[43] R. N. Kulkarni , K. Almind , H. J. Goren , J. N. Winnay , K. Ueki , T. Okada , C. R. Kahn , Diabetes 2003, 52, 1528. 12765966 10.2337/diabetes.52.6.1528

[44] A. Hammoutene , P. E. Rautou , J. Hepatol. 2019, 70, 1278. 30797053 10.1016/j.jhep.2019.02.012

[45] H. Wang , P. F. Pilch , L. Liu , J. Biol. Chem. 2019, 294, 10544. 31126986 10.1074/jbc.RA119.008824 PMC6615684

[46] V. C. Cogger , M. Mohamad , S. M. Solon‐Biet , A. M. Senior , A. Warren , J. N. O'Reilly , B. T. Tung , D. Svistounov , A. C. McMahon , R. Fraser , D. Raubenheimer , A. J. Holmes , S. J. Simpson , D. G. Le Couteur , Am. J. Physiol.: Heart Circ. Physiol. 2016, 310, H1064. 26921440 10.1152/ajpheart.00949.2015

[47] H. Yokomori , M. Oda , M. Ogi , Y. Kamegaya , N. Tsukada , H. Ishii , Liver 2001, 21, 198. 11422783 10.1034/j.1600-0676.2001.021003198.x

[48] A. Warren , V. C. Cogger , I. M. Arias , R. S. McCuskey , D. G. Le Couteur , Microcirculation 2010, 17, 32. 20141598 10.1111/j.1549-8719.2009.00004.x PMC4309280

