# Supplementary material for: Cavin1 Deficiency Causes Disorder of Hepatic Glycogen Metabolism and Neonatal Death by Impacting Fenestrations in Liver Sinusoidal Endothelial Cells
Source: Adv Sci (Weinh). 2020 Aug 21;7(19):2000963. doi: 10.1002/advs.202000963 (PMC7539207; doi:10.1002/advs.202000963)
Supplement: Supplementary file 1 — Supporting Information [file ADVS-7-2000963-s001.pdf]

WILEY-VCH

© 2020 Wiley-VCH GmbH

ADVANCED SCIENCE
Open Access

Supporting Information

for *Adv. Sci.*, DOI: 10.1002/ advs.202000963

Cavin1 Deficiency Causes Disorder of Hepatic Glycogen Metabolism and Neonatal Death by Impacting Fenestrations in Liver Sinusoidal Endothelial Cells

Zhuang Wei, Jigang Lei, Feng Shen, Yuxiang Dai, Yan Sun, Yilian Liu, Yan Dai, Zhijie Jian, Shilong Wang, Zhengjun Chen, Kan Liao,* and Shangyu Hong**

Supporting Information

Cavin1 deficiency causes disorder of hepatic glycogen metabolism and neonatal death by impacting fenestrations in liver sinusoidal endothelial cells

Zhuang Wei, Jigang Lei, Feng Shen, Yuxiang Dai, Yan Sun, Yilian Liu, Yan Dai, Zhijie Jian, Shilong Wang, Zhengjun Chen, Kan Liao* and Shangyu Hong**

Table S1. The genotype analysis of mice at weaning by crossing *Cavin1*$^{+/-}$ parents

Litter No.	*Cavin1*$^{+/+}$	*Cavin1*$^{+/-}$	*Cavin1*$^{-/-}$	total
Litter 1	1	7	0	8
Litter 2	2	2	0	4
Litter 3	0	4	0	4
Litter 4	3	4	0	7
Litter 5	2	5	0	7
Litter 6	1	3	0	4
Litter 7	1	2	0	3
Litter 8	0	3	0	3
Litter 9	1	2	0	3
Litter 10	1	4	0	5
Litter 11	2	3	0	5
Litter 12	3	2	0	5
Litter 13	2	2	0	4
Litter 14	4	2	1	7
Litter 15	1	2	0	3
Sum	24	47	1	72
O	24	47	1	72
E	18	36	18	72
O-E	6	11	-17	0
$(O-E)^2/E$	2	3.36	16.06	21.42

X^2=21.42, p<0.01, significant difference; O, observed; E, expected.

Table S2. The genotype analysis of newborn pups by crossing *Cavin1*$^{+/-}$ parents

Litter No.	*Cavin1*$^{+/+}$	*Cavin1*$^{+/-}$	*Cavin1*$^{-/-}$	total
Litter 1	1	8	2	11
Litter 2	2	6	1	9
Litter 3	5	6	0	11
Litter 4	2	6	1	9
Litter 5	0	6	1	7
Litter 6	3	2	3	8
Litter 7	3	3	1	7
Litter 8	2	4	2	8
Litter 9	2	4	2	8
Litter 10	2	5	1	8
Litter 11	4	4	1	9
Litter 12	3	5	4	12
Sum	29	59	19	107
O	29	59	19	107
E	26.75	53.5	26.75	107
O-E	2.5	5.5	-7.75	0
$(O-E)^2/E$	0.19	0.57	2.25	3.01

X^2=3.01, 0.1<P<0.25, no significance; O, observed; E, expected.

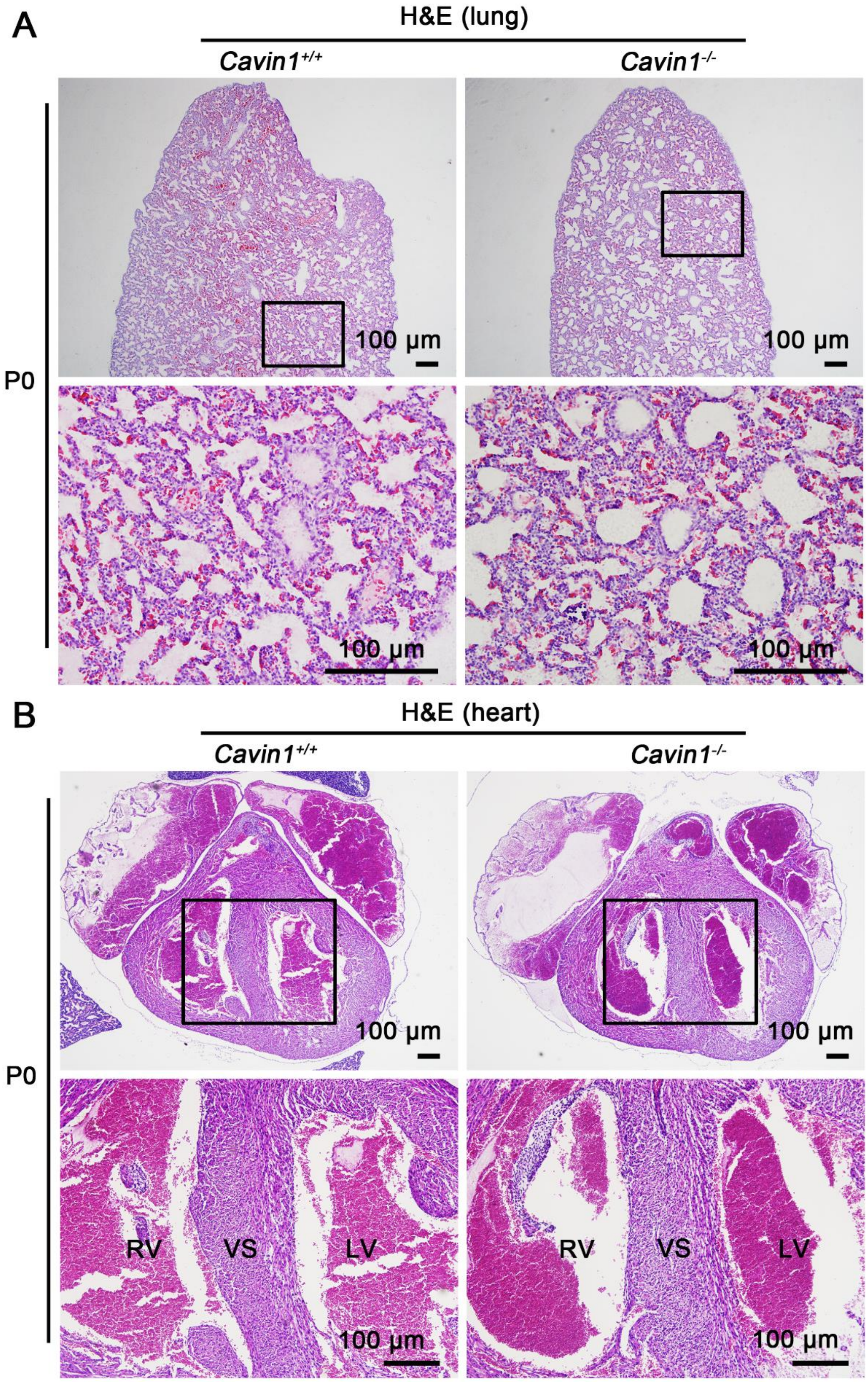

Figure S1. Structures of lung and heart tissue are normal in Cavin1 deficient pups. (A, B) H&E staining of lung and heart tissue from *Cavin1*$^{+/+}$ and *Cavin1*$^{-/-}$ newborn pups at P0 stage. RV, right ventricle; LV, left ventricle; VS, ventricular septum.

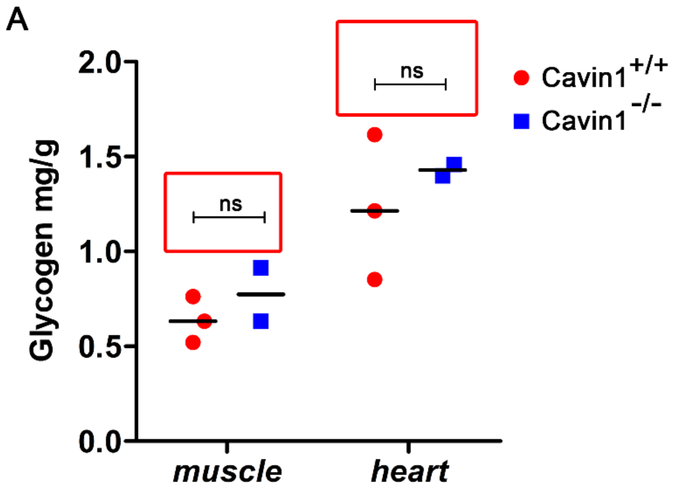

Figure S2. Cavin1 deficiency does not affect glycogen in skeletal muscle and heart. (A) Glycogen in skeletal muscle and heart of normally fed adult *Cavin1*$^{+/+}$ or *Cavin1*$^{-/-}$ homozygous mice. ns, not significant; n=2~3 mice each group.

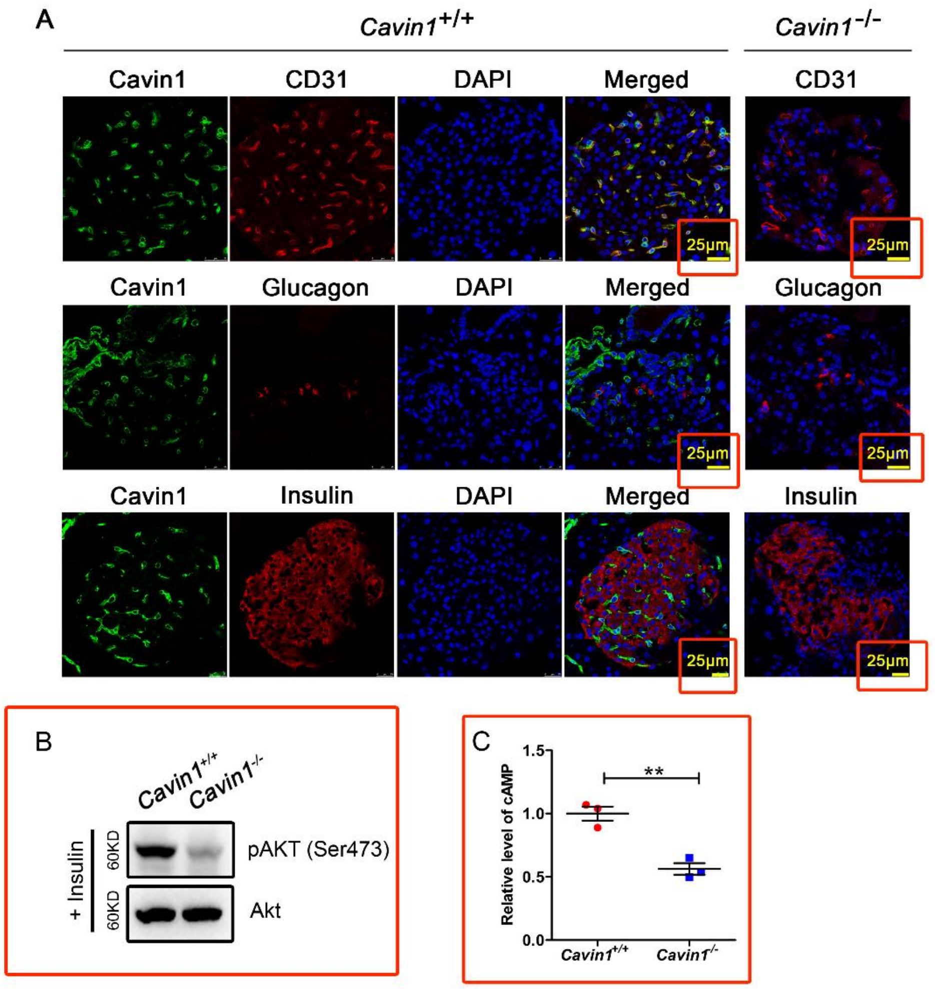

Figure S3. The Cavin1 expression in pancreatic islet.
(A) Pancreatic islets from *Cavin1*$^{+/+}$ or *Cavin1*$^{-/-}$ mice were stained for Cavin1, CD31, insulin or glucagon. Nuclei were stained by DAPI. (B)Phosphorylation of AKT and total AKT levels in the livers of insulin treated *Cavin1*$^{+/+}$ or *Cavin1*$^{-/-}$ mice. (C) The relative cAMP levels in the livers of glucagon treated *Cavin1*$^{+/+}$ or *Cavin1*$^{-/-}$ mice. ** p<0.01; n=3 mice per group.

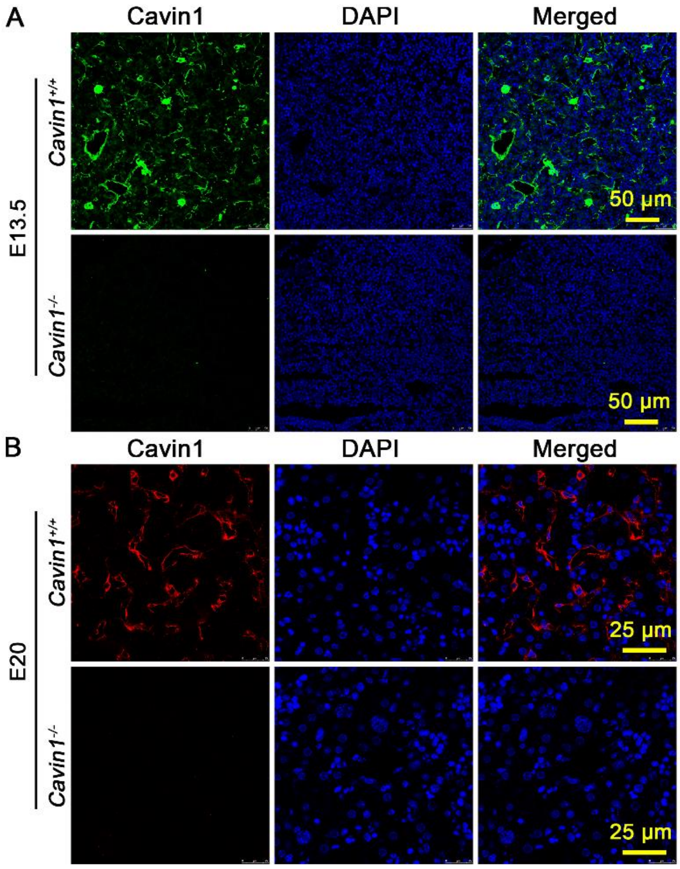

Figure S4. Cavin1 is predominantly expressed in endothelial cells of liver vessels. (A, B) Immunofluorescence staining of Cavin1 in the liver of *Cavin1*$^{+/+}$ and *Cavin1*$^{-/-}$ mice at E13.5 and E20 stages. Nuclei were stained by DAPI.

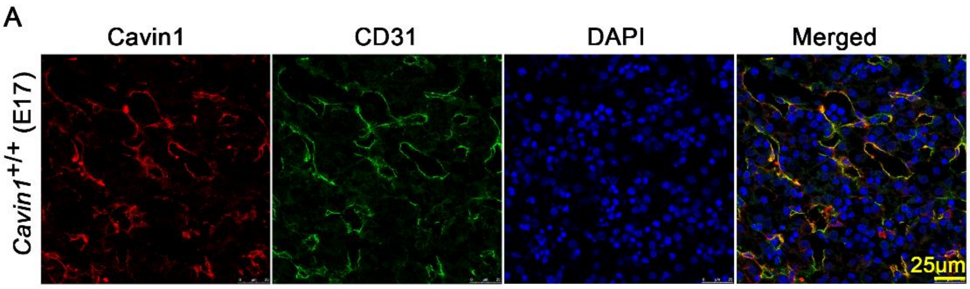

Figure S5. Co-expression of Cavin1 and CD31 in LSECs of E17.5 wild-type fetus.
(A) Livers from wild-type (*Cavin1*$^{+/+}$) mice were stained for Cavin1 and CD31. Nuclei were stained by DAPI.

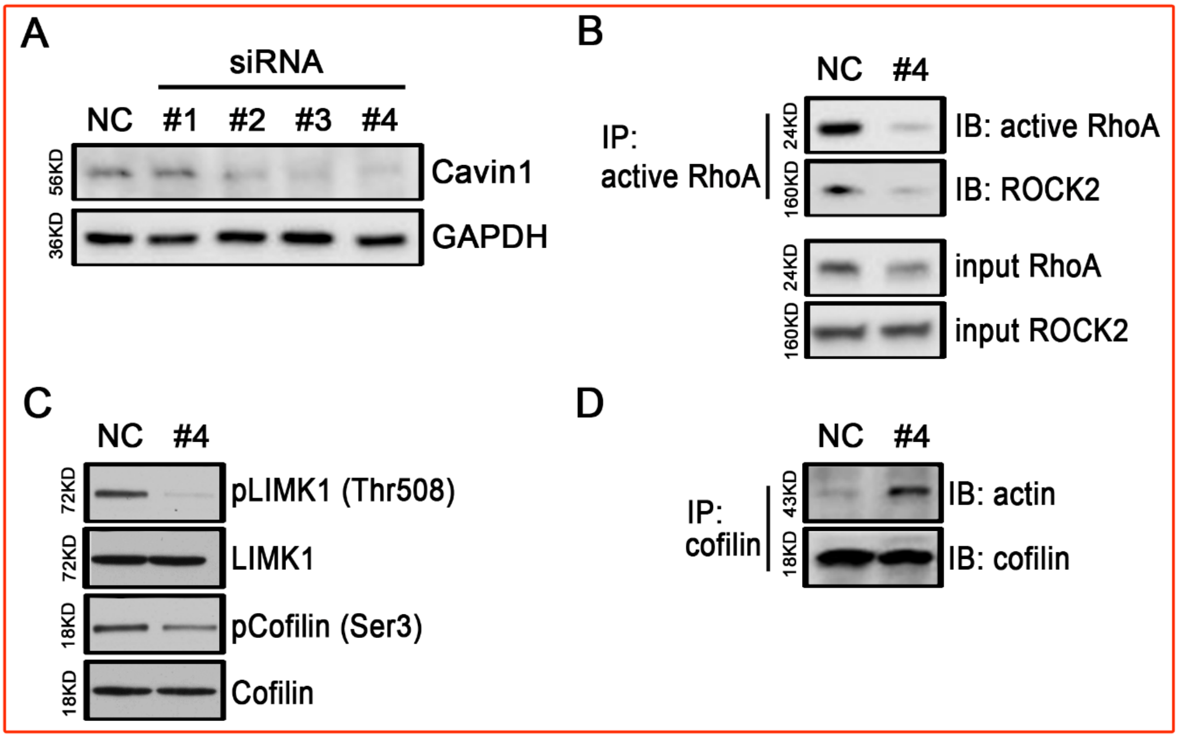

Figure S6. Knocking-down Cavin1in HUVECs inhibited the dynamics of the actin cytoskeleton.
(A) The Cavin1 expression in siRNAs treated HUVECs. (B) Western blot analysis of active Rho A and its binding with ROCK2 in the Cavin1-knockdown HUVECs. Input Rho A, input ROCK2 and HSP90 are shown as control. (C) Western blot analysis of phosphorylated LIMK1 at Thr508 and phosphorylated cofilin at Ser3 in the Cavin1-knockdown HUVECs. (D) Western blot analysis of cofilin co-immunoprecipitation with actin in the Cavin1-knockdown HUVECs.

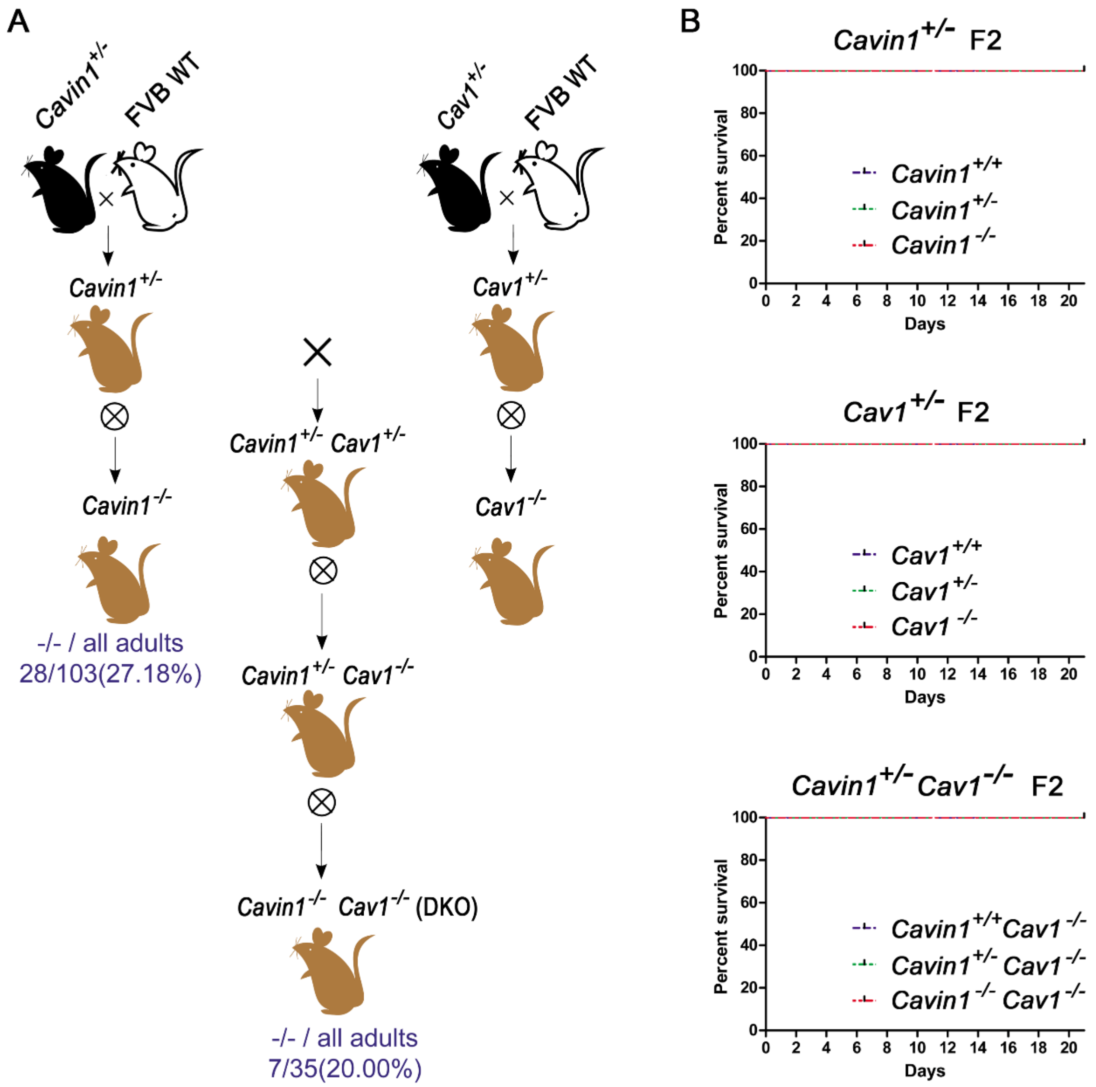

Figure S7. The deletion of Cavin1 does not cause neonatal death in C57BL/6J-FVB mixed background..

(A) The strategy of *Cavin1*, *Cav1* and double knockout mice with C57BL/6-FVB genetic background. × indicates hybridization; × in circle indicates the selfing. (B) The survival rate of newborn pups of *Cavin1*$^{+/-}$, *Cav1*$^{+/-}$ and *Cavin1*$^{+/-}$::*Cav1*$^{-/-}$ (*Cavin1*$^{+/-}$*Cav1*$^{-/-}$) heterozygous parents in C57BL/6-FVB mixed background, respectively.

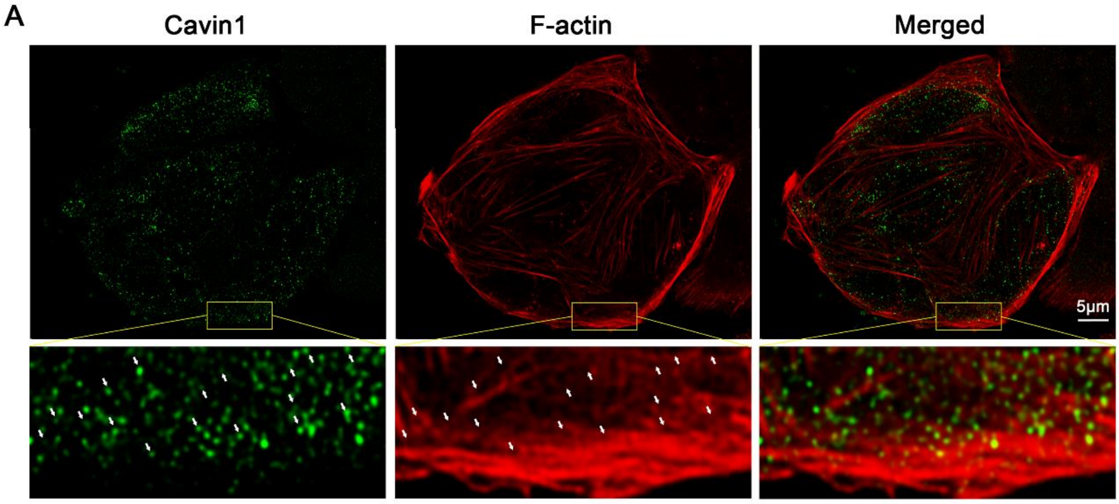

Figure S8. The Cavin1 is not colocalizing with F-actin in LSECs.
(A) Immunofluorescent staining of Cavin1 and F-actin in primary cultured LSECs. The parts in yellow rectangular boxes are enlarged and listed below. The white arrows indicate the Cavin1 localizing in the empty ring area close to F-actin.

Experimental Section

Mice and treatments:

Cavin1 knockout mice (C57BL/6.129S6-Ptrftm1Pfp/J) were purchased from the Jackson Laboratory and back-crossed with wild-type C57BL/6J mice for over 20 generations. Back-crossed heterozygous Cavin1 deficient mice were used as breeding pairs to generate *Cavin1*$^{-/-}$ mice for experiments. All experiments were performed under the ethical guidelines of the Institute of Biochemistry and Cell Biology.

The newborn pups were monitored by real-time camera and their genotypes were detected by PCR analysis. Primer sequences are listed as following: 5'-GAGTCCCGGTGCGCACGGAC-3', 5'-ACCTTGCGCACCTTCTCCAG-3', 5'-TATGCAGCAACGAGACGTCA-3'.

For rescue experiments, newborn pups were intraperitoneally injected with 50 µl of 50% D-glucose solution in phosphate-buffered saline (PBS) or an equal volume of PBS at indicated times. Then, the injected pups were monitored and their survival rate was calculated using GraphPad Prism 5.0 (GraphPad Software Inc, LaJolla, CA, USA).

Measurements of blood glucose and serum glucagon:

Blood was collected from tail tip of pups at 9 hours after birth or fetus at 24 hours before birth, and used to measure blood glucose with a blood glucose meter (SureStep Plus, Johnson). For serum glucagon measurement, blood from heart of pups or fetus was incubated at room temperature for 20 min and centrifuged at 626 g for 20 min. Serum was collected and used to detect serum glucagon level glucagon ELISA kit (Jiancheng, H183).

Histological analysis and immunofluorescence staining:

Liver was fixed in Carnoy's solution containing 60% methanol, 30% chloroform, 10% glacial acetic acid at 4 °C overnight. Subsequently, the samples were hydrated with 100%, 95% and 75% ethanol sequentially for 2 hours in each solution, then dehydrated with 15%, 20% and 30%

glucose solution in PBS for 8 hours in each solution. The rehydrated samples were embedded in paraffin or OCT frozen reagent and sectioned for hematoxylin and eosin (H&E) staining, periodic acid and Schiff's reagent (PAS) staining and immunofluorescence staining. The images of stained sections were randomly captured by light microscopy (BX51, Olympus). The quantitative analysis of PAS-positive area fraction per field in liver was performed using Image J software (NIH Image, Bethesda, MD, USA).

For immunofluorescence staining, briefly, sections were incubated with primary anti-CD4 (Santa Cruz Biotechnology, sc-19641), anti-CD31 (Cell Signaling Technology, 77699S), anti-CD36 (Santa Cruz Biotechnology, sc-9154), anti-Tie2 (Santa Cruz Biotechnology, sc-293414), anti-VEGFR2 (Cell Signaling Technology, #9698) anti-Cavin1 (Abcam, ab78553), anti-Glut1 (Abways, CY5360) and anti-Glut2 (Santa Cruz Biotechnology, sc-9117) antibodies at 4 °C overnight, then with diluted secondary antibody conjugated to Alexa 488 (Jackson, A21202) or Alexa 546 (Jackson, A10036) for 1 hour and counterstained with DAPI (Thermo Fisher, USA) for 10 minutes at room temperature. All images were taken by a laser-scanning confocal microscopy (Leica, Sp8). The area fraction of blood vessels per field in liver were quantitatively analyzed using Image J software (NIH Image, Bethesda, MD, USA).

Western blot:

Total protein of tissues was extracted using the buffer containing 1% sodium deoxycholate, 1% NP-40, 0.2% SDS, 50 mM Tris (pH 6.8), 200 mM NaCl, 2 mM EDTA, 1 mM NaF, 1 mM PMSF and 2 μl/ml protease inhibitors cocktail 1 and 2. Equal amounts of protein were loaded and separated by SDS polyacrylamide gels (EpiZyme, PG112), and subsequently transferred to the PVDF membranes (Millipore, IPVH08130). The membranes were blocked with 5% (W/V) milk for 1 hour at room temperature, then incubated with diluted primary antibodies against CD4, CD31, CD36, Tie2, VEGFR2, Glut1, Glut2, pGSK3β (Ser9) (Santa Cruz Biotechnology, sc-11757), GSK3α/β (Santa Cruz Biotechnology, sc-7291), pGS (Ser641, Cell Signaling

Technology, 3891S), GS (Cell Signaling Technology, 3893S), collagen Ⅳ α1 (Abgent, AP7369A), laminin β1 (Proteintech, 23498-1-AP) and GAPDH (Abways, AB2000) at 4 °C overnight. After washing three times with TBS-T, the membranes were incubated with diluted HRP-conjugated secondary antibodies (Sigma-Aldrich, AP307P; Santa Cruz Biotechnology, sc-516102) for 1 hour at room temperature. The protein bands were detected using the ECL reagent (GE Healthcare, RNP3004).

Transmission and scanning electron microscopy (TEM, SEM):

The liver was cut into small pieces with a razor blade and fixed in 2.5% glutaraldehyde and then in 1% osmium. Dehydration was carried out by successive incubation with 50%, 75%, 80%, 95% and 100% ethanol. For TEM, the sample was further dehydrated with acetone twice and embedded in resin. The thin sections were stained with uranyl acetate and lead citrate for TEM observation. To explore the fenestrae of liver sinusoidal endothelial cells (LSECs) by SEM *in vitro*, primary LSECs were isolated and cultured as previously described[1]. For SEM, the samples after critical point drying were ion-sputtered using a carbon coater (Leica microscope system). The number of fenestrae per μm^2 in liver sinusoidal endothelial cells was quantitatively analyzed by Image J software (NIH Image, Bethesda, MD, USA).

Histamine or nicotine treatment:

8-week-old mice were fed for 12 hours, then intraperitoneally injected with histamine (40 mg/kg body weight) or nicotine (1.967 mg/kg body weight). Subsequently, these mice were fasted for 6 hours and sacrificed for detecting the level of liver glycogen. For glycogen storage experiments, 8 weeks old wild type C57BL/6J mice were fasted overnight and then injected histamine (40 mg/kg body weight) or nicotine (1.967 mg/kg body weight) or PBS control 1 hour prior to re-feeding. 6 hours after the re-feeding, the mice were sacrificed the hepatic glycogen in the liver were analyzed.

The induction of fenestrae formation by latrunculin A treatment:

The formation of fenestrae in the LSECs was induced by latrunculin A as previously reported[2]. Briefly, isolated LSECs were cultured in RIPA1640 medium supplemented with 10% fetal bovine serum and 2 μg/ml vascular endothelial growth factor A (VEGFA). At 70% confluence, 250 ng/ml latrunculin A was added to RIPA1640 medium for 3 hours to induce fenestrae.

Glycogen synthase activity assay:

Liver was collected and weight, then homogenized in lysis buffer containing 20 mM HEPES (pH7.4), 1 mM EDTA, 2 mM $MgCl_2$, 1 mM DTT, 1 mM PMSF and 2 μl/ml protease inhibitors cocktail 1 and 2. 5 μl of liver homogenates was added to 200 μl reaction buffer (1% glycogen, 10 mM glucose-6-phosphate, 3.8 mM uridine 5'-diphosphoglucose, 3.5 mM NADH, 40 mM phospho(enol)pyruvate, 50 units/ml pyruvate kinase, 50 units/ml lactic dehydrogenase in lysis buffer). And the value of OD_{340} was measured every 1 minute for 30 minutes. The relative glycogen synthase activity was calculated from the linear range and normalized by tissue weight.

Glycogen phosphorylase activity assay

Liver was collected and weighed, then homogenized in 1 ml lysis buffer. Subsequently, glycogen phosphorylase activity was determined using glycogen phosphorylase activity assay kit (Solarbio, MS3601) according to the manufacturer's instructions. And the value of OD_{340} was measured every 5 minutes for 10 minutes. The relative glycogen phosphorylase activity was calculated and normalized by tissue weight.

Glucose uptake assay:

Mice were weighed and intraperitoneally injected with 2 g/kg body weight of 50% D-glucose which mixed ^{3}H-labeled glucose (PerkinElmer, Germany) at the volume ratio of 10:1. After 1 hour, hepatic glycogen was extracted by 10% trichloroacetic acid (TCA) solution and the radioactivity was detected by liquid scintillation counter (Beckman Coulter, Chaska, USA).

RhoA activity assay:

RhoA activity assay was performed using RhoA activity assay kit (NewEast, 80601) according to the manufacturer's instructions. Briefly, fresh liver was homogenized in lysis buffer. The lysates were centrifuged at 11800 g for 10 min at 4 °C. Equal amounts of protein was diluted to 1 ml of lysis buffer on ice, and incubated with anti-RhoA-GTP antibodies or normal IgG for 2 hours at 4 °C. Subsequently, the solution were incubated with protein A/G agarose beads for 1.5 hour at 4 ℃, and centrifuged at 3913 g for 1 min at 4 °C. The beads were rinsed by lysis buffer for three times, and eluted with Laemmeli's SDS buffer.

Insulin and glucagon signaling pathway assay:

Mice were fasted for 4 hours and then i.p. injected 10U/g bodyweight insulin or 10mg/kg bodyweight glucagon. 15 minutes after the injection, the mice were sacrificed and the livers wre collected for pAKT western blot and cAMP assay.

Endothelial permeability assay:

Mice were anesthetized and placed on the stage of the upright microscope. Open the abdominal cavity, find the portal vein, and intubate with the indwelling needle. Cut the IVC above the liver to make a gap. And clip the inferior vena cava to the lower part of the liver, so that the portal vein blood flow can only flow unidirectionally into the vein of the heart and out of the gap. Infuse 10ml of PBS + 2mM EGTA solution at 20cm water colum pressure (2 drops/s speed).

Then, infuse 2ml 0.5mg/ml dextran-FITC at the same rate. At this time, the fluorescent signal on the liver surface is imagined with a 20X water lens. Wash the dextran-FITC by infusing 10ml of PBS + 2mM EGTA solution at the same speed. After a few seconds, the dextran-FITC in the blood vessel will be washed away. At this time, the fluorescent signal were imagined again. The collected images were analyzed for fluorescence density using Image Pro Plus.

Single-cell RNA sequencing data analysis:

Single cell data was obtained from (GSE129516). Seurat package were used for standardizing the data and for identification of highly variable features. Then, data was rescaled according to cell cycle related genes. Then use principal component analysis (PCA) to reduce the dimension. Next, Find the neighbors of each cell by embedded K-nearest neighbor (KNN) graph, and then use the Louvain algorithm to cluster the cells, and then project the results of the clustering on the dimension reduction results from embedded tSNE (t-Distributed Stochastic Neighbor Embedding) and Umap (Uniform Manifold Approximation and Projection). Mark each cell population with known markers.

HUVECs culture and siRNA treatment:

HUVECs were cultured in endothelial media with growth factor (EGM2 from Promocell) and antibiotics. 4 siRNAs (5‘-3‘: CCAGAUCCAGCUGACUCAATT; GCCGCAACUUUAAAGUCAUTT; GGAGGUUGAGGAGGUUAUUTT; UCUACAAGGUGCCACCCUUTT) ordered from GenePharma Int., were transfected into the cells with lipofectamine 2000. 72 hours after the transfection, the cells were collected for further investigation.

Primary hepatocyte culture and treatment:

The primary hepatocytes were isolated from *Cavin1*$^{+/+}$ and *Cavin1*$^{-/-}$ mice, and cultured in low glucose DMEM with 10% FBS and antibiotics. Once attached with the plate, the cells were serum starved for 2 hours before treated with 100nM glucagon or 100nM insulin. After 6 hours treatment, the glycogens in the cells were measured and analyzed.

Statistical analysis:

The data were analyzed with GraphPad Prism 5.0 (GraphPad Software Inc, LaJolla, CA, USA) and expressed as the mean ± SD. Mann Whitney test was used for statistical comparisons between two groups. One-way analysis of variance (ANOVA) followed by post hoc Newman-Keuls test was used for statistical comparisons between multiple groups. Two-way ANOVA was used for grouped analysis. P values of < 0.05 were considered statistically significant. *$P<0.05$, **$P<0.01$, ***$P<0.001$; ns, not significant.

References

[1] G. W. Daneker, S. A. Lund, S. W. Caughman, R. A. Swerlick, A. H. Fischer, C. A. Staley, E. W. Ades, In Vitro Cell Dev Biol Anim 1998, 34, 370.

[2] F. Braet, R. De Zanger, D. Jans, I. Spector, E. Wisse, Hepatology 1996, 24, 627.
